# 3,3′-Diindolylmethane Ameliorates Metabolism Dysfunction-Associated Fatty Liver Disease via AhR/p38 MAPK Signaling

**DOI:** 10.3390/nu17101681

**Published:** 2025-05-15

**Authors:** Jiewen Su, Heng Fang, Yunfeng Lin, Yilu Yao, Yanxi Liu, Yuquan Zhong, Xudong Li, Siyu Sun, Bing Huang, Guangyu Yang, Wenxue Li, Yan Zhang, Juntao Li, Jinyin Wu, Weiwen Liu, Qiansheng Hu, Wei Zhu

**Affiliations:** 1 School of Public Health, Sun Yat-Sen University, Guangzhou 510275, China; su18819747726@163.com (J.S.); fangheng2022@163.com (H.F.); 2 Department of Scientific Research, Guangzhou Center for Disease Control and Prevention (Guangzhou Health Supervision Institute), Guangzhou 510405, China; lxdnutrition@163.com (X.L.); gzcdc_huangb@gz.gov.cn (B.H.); gzcdc_yanggy@gz.gov.cn (G.Y.); gzcdc_liwx@gz.gov.cn (W.L.); gzcdc_zhangy@gz.gov.cn (Y.Z.); gzcdc_lijt@gz.gov.cn (J.L.); gzcdc_wujy@gz.gov.cn (J.W.); gzcdc_liuww@gz.gov.cn (W.L.); 3 School of Public Health, Guangdong Pharmaceutical University, Guangzhou 510006, China; linyunfeng2020@163.com; 4 School of Basic Medicine and Public Health, Jinan University, Guangzhou 510632, China; yilu0701131@163.com; 5 School of Public Health, Guangzhou Medical University, Guangzhou 511436, China; liuyanxitcm@163.com (Y.L.); yuquan_zhong@163.com (Y.Z.); 6 School of Public Health, Southern Medical University, Guangzhou 510515, China; sunsiyu8@163.com

**Keywords:** 3,3′-Diindolylmethane, MAFLD, aryl hydrocarbon receptor, p38 MAPK, CD36

## Abstract

**Background/Objectives**: Metabolic dysfunction-associated fatty liver disease (MAFLD) is a chronic hepatic condition marked by lipid buildup, lipotoxicity, and inflammation. Prior research indicates that 3,3′-Diindolemethane (DIM), a natural indole-type phytochemical that is abundant in brassicaceae vegetables, has been reported to reduce body weight and improve lipid metabolism in mice subjected to a high-fat diet (HFD). The aryl hydrocarbon receptor (AhR), a nuclear receptor implicated in lipid metabolism and immune regulation, serves as a functional receptor for DIM. However, the underlying signaling pathways that regulate MAFLD remain elusive. Our objective is to ascertain the beneficial impact of DIM on MAFLD and the associated mechanisms. **Methods**: Hematoxylin and eosin staining, together with Oil Red O staining, were utilized to assess the pathological changes and lipid deposition in the liver. Biochemical analysis was employed to measure levels of triglyceride (TG), total cholesterol (TC), free fatty acid (FFA), aspartate transaminase (AST), alanine transaminase (ALT), low-density lipoprotein cholesterol (LDL-C) and high-density lipoprotein cholesterol (HDL-C). The cell survival rate of HepG2 cells treated with palmitic acid (PA) and DIM was assessed using the CCK-8 assay. Flow cytometry was employed to measure the fluorescence intensity emitted by lipid droplets within cells. Western blotting analysis was performed to assess AhR pathway and fatty acid transporter expression levels in hepatic tissue. **Results**: Our results showed that DIM significantly attenuated body weight gain and hepatic injury brought on by HFD, decreased lipid droplet accumulation in HepG2 cells, and effectively suppressed the phosphorylation of p38 MAPK and the protein expression levels of fatty acid transporters CD36 and FATP4. **Conclusions**: DIM reduced lipid accumulation by activating AhR and suppressing p38 MAPK phosphorylation, thereby inhibiting fatty acid transport and inflammatory responses. These findings suggest that DIM may represent a promising therapeutic candidate for MAFLD, warranting further exploration for clinical applications.

## 1. Introduction

Metabolic dysfunction-associated fatty liver disease (MAFLD) is a prevalent chronic hepatic condition, affecting 25% of the global population [1], and its incidence is still increasing due to lifestyle changes and the rising incidence of obesity [2]. Although numerous studies support the treatment of MAFLD, there are currently no medications explicitly sanctioned for this ailment [3,4]. According to studies, foods and natural plants include phytochemicals called indoles and flavonoids that have lipid-modifying, antioxidant, and anti-inflammatory effects [5]. 3,3′-Diindolylmethane (DIM) is synthesized through the polymerization of its precursor indole-3-carbinol (I3C) in gastric juice. I3C is a naturally occurring indole compound derived from glucosinolates, which are prevalent in cruciferous vegetables such as celery and carrots, via myrosinase hydrolysis [6,7]. Studies have confirmed that DIM or I3C has a variety of biological functions, including anti-oxidation, anti-inflammation [8,9], anti-microbial [10], anti-angiogenesis, and tumor inhibition [11]. The regulation of lipid metabolism and can effectively treat obesity [12]. The investigation into indole compounds exhibiting therapeutic potential represents a crucial advancement in the development of viable pharmaceuticals for addressing chronic noncommunicable diseases, including obesity and MAFLD [13,14].

Aryl hydrocarbon receptor (AhR), a receptor ligand extensively expressed in cells, activates transcription factors and functions to regulate energy metabolism and inflammatory diseases. Many compounds, notably cruciferous vegetable-derived indoles like I3C and DIM, bind to AhR with great affinity. This combination enables these compounds to participate in a variety of biological functions within the body, including energy metabolism, immune regulation, and xenobiotic metabolism [15,16]. AhR is a possible target for the intervention and therapy of liver disease since it is extensively expressed in the liver and controls the immunological and inflammatory responses of the liver [17]. In mice, AhR has been demonstrated to phosphorylate the downstream target transcription factors of the p38 MAPK signaling cascade, including *elk-1*, *creb*, and *atf-2*, indicating that AhR is essential for MAPK activation [18,19]. p38 mitogen-activated protein kinase (p38 MAPK) is serine/threonine mitogen-activated protein kinase; it is expressed in various cells [20]. Studies conducted in vitro have confirmed that AhR has a function in triggering the activation of p38 MAPK. Apoptosis, the cell cycle, and the inflammatory response are all tightly linked to the latter [21]. Studies have shown that the downstream molecule ATF-3 is significantly increased after p38 phosphorylation in mice subjected to HFD, causing liver lipid buildup, inflammation, and fibrosis [22,23]. Studies have shown that increased phosphorylation of p38 MAPK promotes cancer cell growth [24], whereas the advancement of MAFLD may result in liver cancer development. Increasing phosphorylation of p38 MAPK diminishes DIM’s efficacy in cancer treatment [25]; thus, investigating the impact of DIM on p38 MAPK is of considerable practical importance for ameliorating MAFLD.

This study investigates the hepatoprotective effect of DIM on HFD-induced MAFLD mice in vivo and PA-induced lipid-accumulation cells in vitro. We further elucidated the underlying mechanism to show that DIM alleviated MAFLD by reducing p38 MAPK phosphorylation and reducing fatty acid transport.

## 2. Materials and Methods

### 2.1. Drugs and Dosages

Previous studies [26,27,28] have demonstrated that, in population-based experiments, the intake dose of the DIM purifying agent can reach up to 300 mg/day. Based on the equivalent dose conversion coefficient, this level of human intake is approximately equivalent to a DIM intake of 50 mg/kg/day in mice. Furthermore, according to the pharmacokinetic results reported in prior studies, DIM exhibits excellent tolerance at a single dose as high as 300 mg, with its maximum plasma concentration reaching stability [28]. This indicates that the pharmacokinetic characteristics of DIM become saturated at this dose. Consequently, in our animal experiments, we selected a DIM dose of 50 mg/kg/day.

### 2.2. Animals

From Guangdong Medical Laboratory Animal Center, thirty male C57BL/6J mice weighing 18–20 g and aged 6–8 weeks were acquired. All animal research received approval from the Animal Care and Use Committee of GZCDC (protocol number: GZCDC-ACUC-2023057). A 12 h light: 12 h dark cycle, 22 ± 2 °C temperature, and 40–70% humidity were all features of the specified pathogen-free (SPF) environment in which the mice were kept. Following 7 days of domestication, the mice were randomized to 3 sets: A group of mice bred on a normal control diet (NCD, *n* = 10, 10 kcal% of fat, 3.85 kcal/g diet), a group fed a high-fat diet (HFD, *n* = 10, 60 kcal% of fat, 5.24 kcal/g diet), and a group of mice fed HFD was given 50 mg/kg/day of DIM (Sigma-Aldrich, St. Louis, MO, USA, HFD + DIM, *n* = 10) orally via gastro. A 0.5% sodium carboxymethylcellulose solution was used to dissolve DIM, and both the NCD and HFD groups obtained an equivalent capacity of the 0.5% sodium carboxymethylcellulose solution. Sixteen weeks later, mice were humanely euthanized and specimens of tissue and blood were collected for subsequent in-depth examination.

### 2.3. Biochemical Analysis

Total cholesterol (TC), triglycerides (TG), and free fatty acids (FFA) were measured after liver tissue had been homogenized in 95% ethanol and the supernatant was collected. Serum concentrations of TC, TG, FFA, alanine aminotransferase (ALT), aspartate aminotransferase (AST), high-density lipoprotein cholesterol (HDL-c), and low-density lipoprotein cholesterol (LDL-c) were quantified utilizing kits supplied by Jiancheng BioEngineering Institute (Nanjing, China).

### 2.4. Histopathological Analysis

Livers were preserved in paraformaldehyde at 4%. Services for oil red O (ORO) staining and hematoxylin and eosin (H&E) staining were rendered by Servicebio (Wuhan, China). Images were captured using a Leica light microscope (Wetzlar, Germany). ORO staining was used for quantitative analysis using Image J software (Version 1.53t, Bethesda, MD, USA).

### 2.5. Cell Culture and Cell Treatment

HepG2 (ATCC, Manassas, VA, USA), a human hepatocellular carcinoma cell line, was cultured in DMEM Medium (Gibco, Grand Island, NY, USA) with 1% penicillin-streptomycin and 10% fetal bovine serum (FBS) added. At 37 °C, the culture was kept in a humidified incubator with 5% CO_2_. Four groups of cells were created in order to cause excessive lipid accumulation in an in vitro model of hepatic steatosis: (1) control group (CON), (2) palmitic acid (Sigma-Aldrich, St. Louis, MO, USA) stimulation model group (PA), (3) model group containing DIM (PA + DIM), (4) model group containing DIM and CH223191 (MCE, Monmouth Junction, NJ, USA) (PA + DIM + CH223191). All cell groups, with the exception of the control group, were exposed for 24 h to media supplemented with 0.3 mM PA. For the final 24 h of the 48 h treatment, cells were treated with DIM after they had been dissolved in DMSO (Sigma-Aldrich, St. Louis, MO, USA).

### 2.6. CCK-8 Assay

HepG2 cells (1 × 10^4^ cells/mL) were seeded into 96-well plates and cultured for 24 h under standard conditions. Following incubation with PA at concentration gradients of 0, 25, 50, 100, 200, 400, 800, and 1600 μM, or DIM at concentration gradients of 0, 10, 20, 40, and 80 μM, the culture medium was aspirated. For inhibition experiments, each well was filled with 10% CCK-8 (Beyotime, Shanghai, China) solution, which was then incubated at 37 °C for around 30 min. BioTek microplate reader (Biotek, Winooski, VT, USA) was then used to evaluate cell viability at a wavelength of 450 nm. The formula of cell viability (%) = [(experimental well-blank well)/(control well-blank well)] × 100% was used to analyze cell viability.

### 2.7. Oil Red O Staining

Following therapy, the HepG2 cells were gently washed twice with PBS before being fixed for 15 min at room temperature with paraformaldehyde at 4%. Subsequently, the cells underwent differentiation with 60% isopropanol for 1 min and were dyed using Oil Red O solution (Solarbio, Beijing, China, prepared by mixing saturated Oil Red O and deionized water in a 3:2 ratio). Before being examined and photographed under a light microscope, the cells were first incubated for 20 min at 37 °C, then eluted for 1 min with 60% isopropanol, and lastly, rinsed with 1 mL PBS.

### 2.8. Flow Cytometry

The culture medium was removed from the 6-well plate containing the treated HepG2 cells and washed twice with PBS at 37 °C. An equal volume of 2.5 μM Bodipy 493/503 (GLPBIO, Montclair, CA, USA) staining working solution was added to each well, followed by incubation in a light-protected environment at 37 °C for 30 min. Subsequently, the cells were rinsed with PBS three times in a gentle manner. The cells were scraped off and centrifuged for five minutes at 1200 rpm and the resulting cell pellets were reconstituted in 500 μL of PBS to create the cell suspension. Flow cytometry (Agilent Technologies, Santa Clara, CA, USA) was then used to assess the fluorescence intensity in the FITC channel.

### 2.9. Western Blotting

Radioimmunoprecipitation assay (RIPA) buffer (KeyGEN BioTECH, Nanjing, China) was used to extract total proteins, including protease and phosphatase inhibitors and phenylmethylsulfonyl fluoride (PMSF). The BCA protein assay kit (KeyGEN BioTECH, Nanjing, China) was utilized in order to produce all extracts and measure the protein quantities. SDS-PAGE was used to resolve 40 µg of extracted proteins, which were then transferred to PVDF membranes (Millipore, Billerica, MA, USA). The membranes were then blocked using either 5% skim milk or bovine serum albumin for two hours at room temperature. After that, the primary antibodies were kept at 4 °C for the whole night. The antibodies utilized are enumerated in Table S1. Secondary antibodies were then incubated on the membranes for 2 h at room temperature. The protein bands were visualized using an ECL chemiluminescence detection kit (Abclonal, Wuhan, China) and further analyzed with an imaging system (Analytik Jena, Jena, TH, Germany) and quantified using Image J software (Bethesda, MD, USA).

### 2.10. Statistical Analysis

The experimental data were expressed as mean ± standard deviation ($\bar{\chi }$ ± SD) and the software named SPSS (Version 25, Chicago, IL, USA) was used to conduct statistical analyses. One-way analysis of variance (ANOVA) was used for multiple group comparisons, and the LSD-*t* test was used for analyzing measurement data that was normally distributed. To create the graphs, GraphPad Prism (Version 10; La Jolla, CA, USA) was used. *p*-values below 0.05 were considered statistically significant.

## 3. Results

### 3.1. DIM Ameliorated Obesity and Liver Damage Induced by HFD

**Figure 1:**
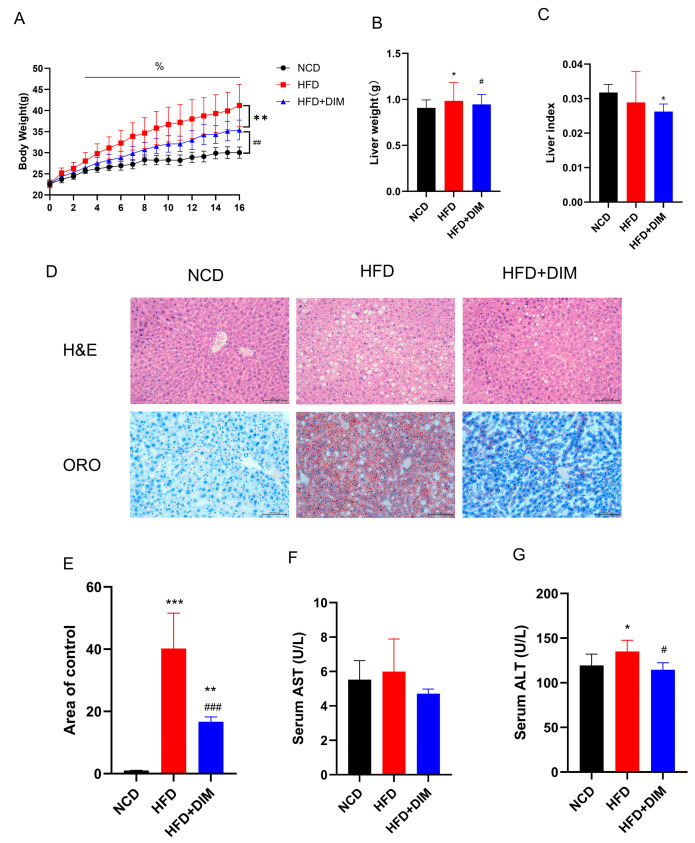
DIM attenuated body weight and alleviated liver injury in mice. (**A**) Trends in mouse body weight. (**B**) Differences in live weight. (**C**) Differences in live index. (**D**) The H&E and ORO images clearly and precisely illustrate the percentage of hepatocyte steatosis; (**E**) Quantitative analysis of ORO images. (**F**) Differences in serum AST. (**G**) Differences in serum ALT. * *p* < 0.05, ** *p* < 0.01, *** *p* < 0.001 vs. NCD group, # *p* < 0.05, ## *p* < 0.01, ### *p* < 0.001 vs. HFD group and % *p* < 0.05 indicates differences between groups at various time points.

Throughout the trial duration, the body weight of mice in each cohort was noted to increase progressively. Treatment with DIM was found to effectively mitigate excessive weight gain (*F* = 28.94, *p* < 0.01) of mice and reduce the liver weight (*F* = 3.43, *p* < 0.05), although not significantly reduce the liver index (*F* = 2.50, *p* = 0.10) (Figure 1A–C). As compared with the NCD group, HFD resulted in a significant increase in serum TG (*F* = 4.95, *p* < 0.05), TC (*F* = 5.195, *p* < 0.05), LDL-c (*F* = 9.99, *p* < 0.01) and hepatic TG (*F* = 9.60, *p* < 0.01) levels, and the DIM intervention could significantly reduce serum TG, LDL-c (*p* < 0.05) and hepatic TG (*p* < 0.05), TC (*F* =9.57, *p* < 0.01), FFA (*F* = 2.58, *p* < 0.05) levels and increase HDL-c (*F* = 5.79, *p* < 0.05) levels (Table 1 and Table 2). The extent of liver injury was assessed using H&E staining and ORO staining (Figure 1D,E), and ALT and AST levels in the serum were assessed. (Figure 1F,G). Histopathological analysis demonstrated steatosis, hepatocellular disease and interstitial expansion in mice fed HFD. However, dietary supplementation with DIM improved hepatic steatosis symptoms. Additionally, serum ALT (*F* =4.60, *p* < 0.05) levels were significantly decreased in DIM-treated mice compared to those in HFD-fed mice. Therefore, whereas DIM can reduce lipid droplet accumulation and alleviate liver damage, HFD produces an excessive buildup of lipid droplets in hepatocytes and results in liver injury.

### 3.2. HepG2 Cells Accumulated Lipids as a Result of PA Activation, Which DIM Decreased

**Figure 2:**
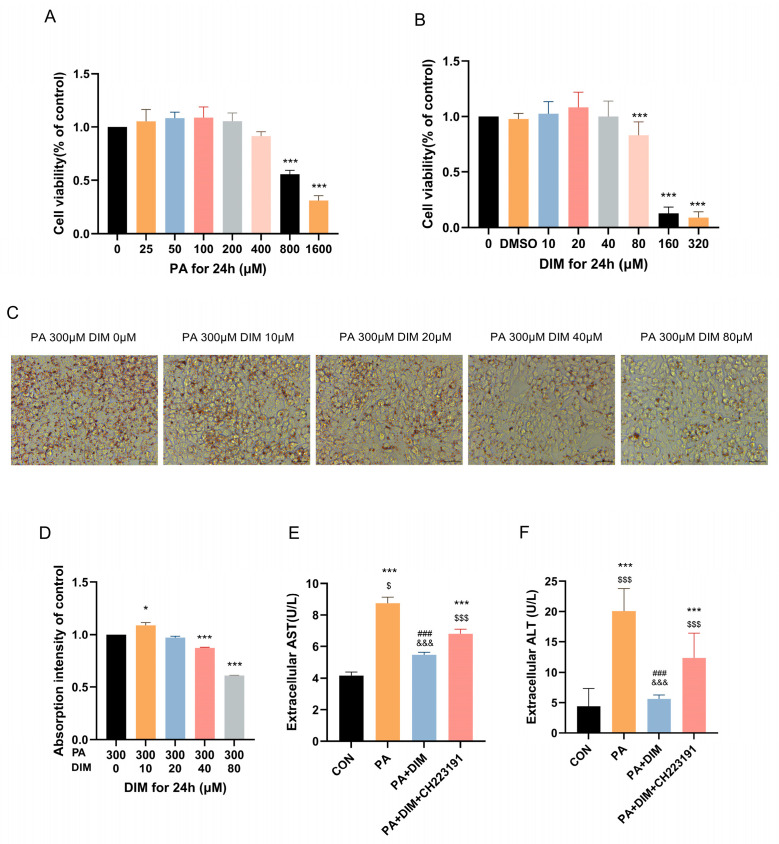
DIM attenuated droplet accumulation in HepG2 cells. (**A**,**B**) The CCK-8 assay was used to evaluate the impact of PA and DIM on the cytotoxicity of HepG2 cells. (**C**) Lipid droplets in HepG2 cells were visualized using ORO staining. (**D**) Analysis of HepG2 cells’ lipid droplets stained with oil red O statistically. (**E**,**F**) DIM’s effect on the AST and ALT levels in the HepG2 cell culture medium. * *p* < 0.05, *** *p* < 0.001 vs. CON group, ### *p* < 0.001 vs. PA group, $ *p* < 0.05, $$$ *p* < 0.001 vs. PA + DIM group, and &&& *p* < 0.001 vs. PA + DIM + CH223191 group.

The impact of different PA (*F* = 88.86, *p* < 0.001) and DIM (*F* = 112.94, *p* < 0.001) on HepG2 cell proliferation was evaluated using the CCK-8 assay. The findings demonstrated that the concentrations employed in the experiments were non-cytotoxic, thereby ensuring the safety of subsequent analyses. In order to achieve cell viability above 90%, we determined the concentration of PA at 300 μM and DIM at 40 μM (Figure 2A,B). ORO staining of HepG2 cells was used to quantitatively evaluate the degree of intracellular lipid accumulation (Figure 2C,D). The results demonstrated that, after the addition of PA-induced the aggregation of intracellular lipid droplets (*F* = 546.72, *p* < 0.01) in HepG2 cells and significantly increased the levels of AST (*F* = 151.10, *p* < 0.01) and ALT (*F* = 15.84, *p* < 0.01) in the culture medium (Figure 2E,F). However, upon the incorporation of DIM, lipid accumulation was attenuated, and AST and ALT levels in the culture medium dropped dramatically. Nevertheless, CH223191 pretreatment dramatically raised AST and ALT levels and greatly encouraged the production of intracellular lipid droplets. These results suggest that palmitic acid stimulation triggers intracellular lipid accumulation in HepG2 cells, that this accumulation is mitigated by the addition of DIM, and that the inhibitory effect of DIM is counteracted by CH223191.

### 3.3. DIM Reduced p38MAPK Phosphorylation in Mice and HepG2 Cells

**Figure 3:**
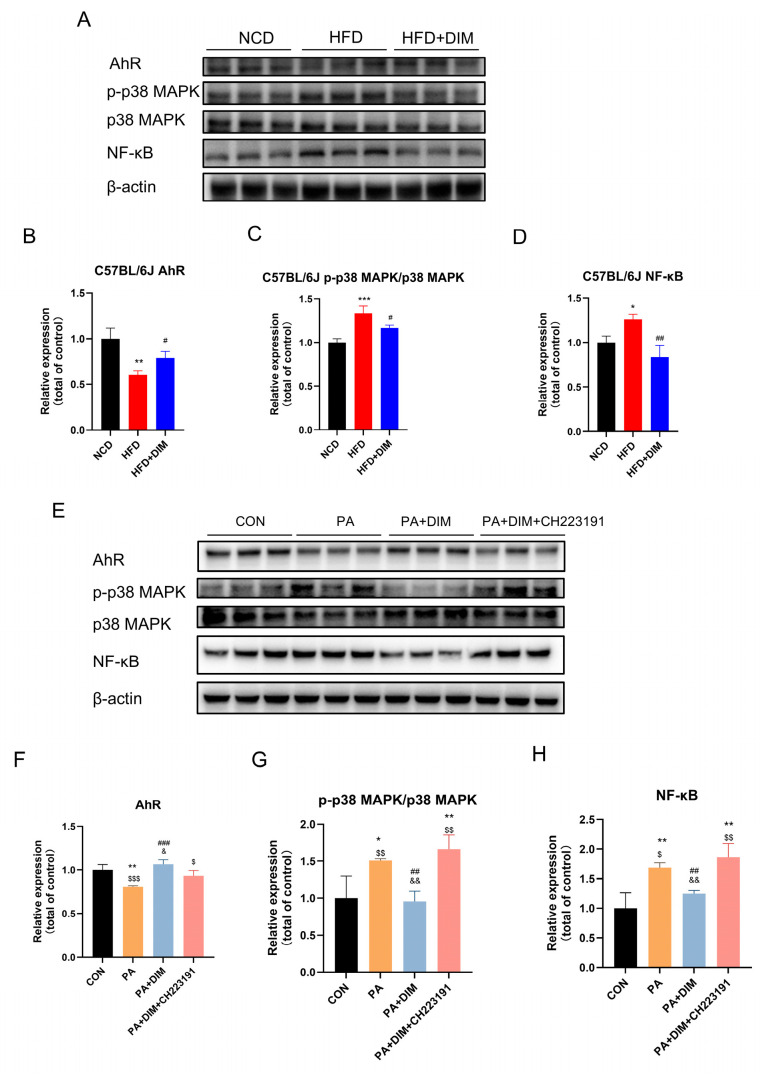
DIM attenuated the phosphorylation of p38MAPK in mice and cells. (**A**–**D**) Western blotting analysis of AhR, p-p38 MAPK, p38 MAPK and NF-kB protein levels in mice and densitometric quantification of them, * *p* < 0.05, ** *p* < 0.01, *** *p* < 0.001 vs. NCD group, # *p* < 0.05, ## *p* < 0.01 vs. HFD group. (**E**–**H**) Western blotting analysis of AhR, p-p38 MAPK, p38 MAPK and NF-kB protein levels in HepG2 cells and densitometric quantification of them, * *p* < 0.05, ** *p* < 0.01 vs. CON group, ## *p* < 0.01, ### *p* < 0.001 vs. PA group, $ *p* < 0.05, $$ *p* < 0.01, $$$ *p* < 0.001 vs. PA + DIM group, and & *p* < 0.05, && *p* < 0.01, vs. PA + DIM + CH223191 group.

Western blotting results of mouse liver tissue showed that AhR (*F* = 16.80, *p* < 0.01) protein expression was decreased, protein expression of p38 MAPK phosphorylation (*F* = 25.58, *p* < 0.01) and NF-κB (*F* = 16.15, *p* < 0.01) were significantly increased in mice treated with HFD (Figure 3A–D). Yet, mice given DIM showed higher levels of AhR protein expression. Both p38 MAPK phosphorylation and NF-κB protein expression were markedly reduced. The protein expression of p38 MAPK phosphorylation (*F* = 10.56, *p* < 0.01) and NF-κB (*F* = 14.83, *p* < 0.01) strongly increased in HepG2 cells treated with PA, while the protein expression of AhR (*F* = 14.05, *p* < 0.05) reduced. (Figure 3E–H). When DIM was added, p38 MAPK phosphorylation and NF-κB dramatically dropped, whereas AhR protein production elevated. Protein expression was similar to the PA group. According to the data above, lipid buildup in the livers of mice and HepG2 cells raised p38MAPK phosphorylation, which DIM therapy decreased.

### 3.4. DIM Attenuated Lipid Transport in Mice and HepG2 Cells

**Figure 4:**
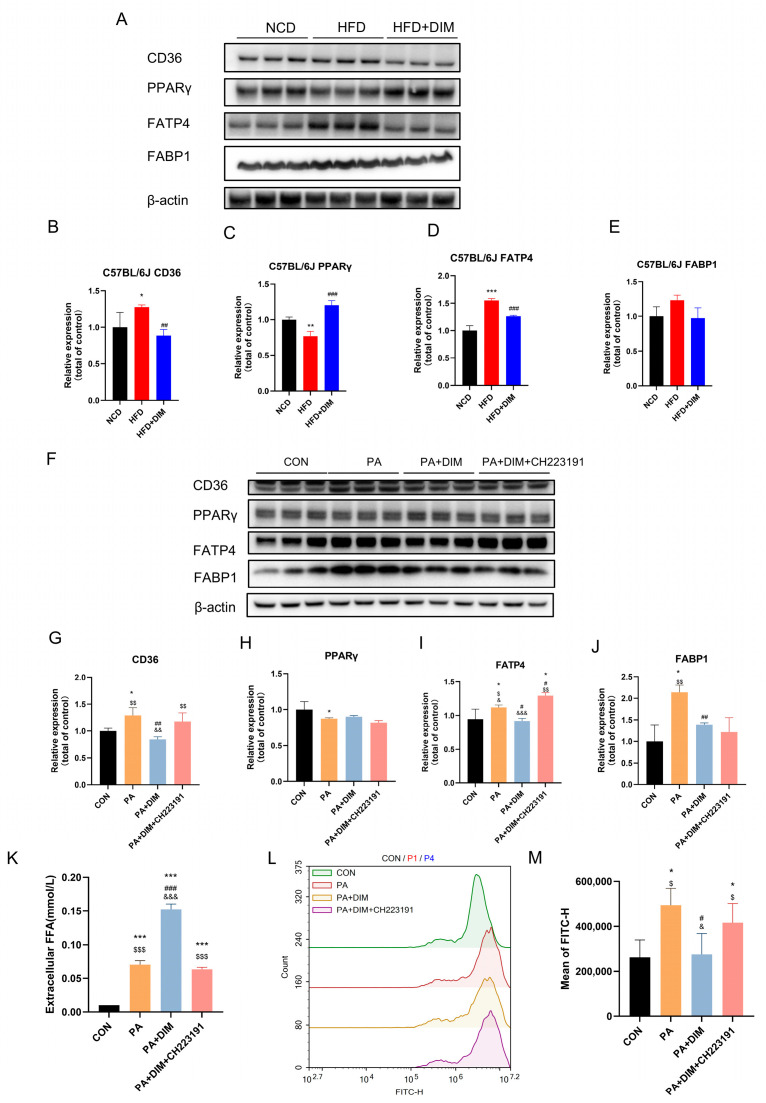
DIM attenuated fatty acid transport in mice and cells. (**A**–**E**) Western blotting analysis of CD36, PPARγ, FATP4 and FABP1 protein levels in mice and densitometric quantification of them, * *p* < 0.05, ** *p* < 0.01, *** *p* < 0.001 vs. NCD group, ## *p* < 0.01, ### *p* < 0.001 vs. HFD group. (**F**–**J**) Western blotting analysis of CD36, PPARγ, FATP4 and FABP1 protein levels in HepG2 cells and densitometric quantification of them; (**K**) DIM’s effect on the FFA level in the HepG2 cell culture medium. (**L**,**M**) Lipid droplets in HepG2 cells were stained with Bodipy 493/503 for visualization. * *p* < 0.05, ** *p* < 0.01, *** *p* < 0.001 vs. CON group, # *p* < 0.05, ## *p* < 0.01, ### *p* < 0.001 vs. PA group, $ *p* < 0.05, $$ *p* < 0.01, $$$ *p* < 0.001 vs. PA + DIM group, & *p* < 0.05, && *p* < 0.01, &&& *p* < 0.001 vs. PA + DIM + CH223191 group.

Since fatty acid transport is essential for lipid droplet generation in the liver, we examined fatty acid transport protein expression. An analysis of Western blotting showed that mice given HFD had dramatically increased CD36 (*F* = 7.64, *p* < 0.05) and FATP4 (*F* = 72.34, *p* < 0.01) protein levels, but PPARγ (*F* = 43.59, *p* < 0.01) protein levels were significantly decreased (Figure 4A–E). However, CD36 and FATP4 protein expression levels were significantly decreased in mice treated with DIM. There was no significant difference in FABP1 (*F* = 2.64, *p* = 0.15) protein expression among the three groups. The protein expression levels of CD36 (*F* = 9.11, *p* < 0.01), FATP4 (*F* = 14.00, *p* < 0.01) and FABP1 (*F* = 10.08, *p* < 0.01) were increased, but the protein expression level of PPARγ (*F* = 4.85, *p* < 0.05) was notably downregulated in HepG2 cells induced by PA (Figure 4F–J). The protein expression levels of CD36, FATP4, and FABP1 were significantly reduced upon the addition of DIM, whereas the expression levels of CD36 and FATP4 were significantly enhanced following the administration of CH223191 but FABP1 (*p* > 0.05) protein expression level did not change significantly. There was no significant difference in the expression of PPARγ (*p* > 0.05) protein among the other two groups. The above data suggest that fatty acid transport is increased in the liver of HFD-fed mice and PA-stimulated HepG2 cells, that DIM can reduce fatty acid transport, and that DIM can block the effect ofPA. After 24 h of therapy, FFA levels in the cell culture media were evaluated to see if DIM improves fatty acid absorption (Figure 4K). The PA group’s FFA (*F* = 391.64, *p* < 0.001) content was much lower than the PA + DIM group’s, indicating that PA was transferred into the cells from the culture media, whereas DIM treatment inhibited the transport of FFAs into the cells. CH223191, a specific inhibitor of DIM, effectively blocked the inhibitory effect of DIM on fatty acid transport (Figure 4L,M). Bodipy493/503, which can permeate the cell membrane and specifically bind to intracellular lipid droplets, was used to reflect the cellular uptake of fatty acids. Findings indicated that the PA group had the maximum fluorescence intensity (*F* = 7.39, *p* < 0.01), while DIM significantly reduced the cellular transport of fatty acids, as evidenced by a marked decrease in fluorescence intensity.

## 4. Discussion

MAFLD is a complex disorder marked by hepatocyte lipid buildup that results in fibrosis, inflammation, and steatosis [29]. Dysregulated lipid metabolism, chronic inflammation, and oxidative stress are all closely related to the pathophysiology of MAFLD [30]. After mice were fed on a high-fat diet to induce MAFLD, the HFD group’s body weight increased by almost 20% compared to the usual chow diet. The MAFLD mouse model was established successfully. As in prior studies, HFD-induced obese mice had a higher liver index [31]. In this study, the HFD group’s liver weight increased, but less than body weight in mice. This study confirmed that HFD increased blood and hepatic TG, LDL-c and HDL-c levels and decreased HDL-c [32]. DIM may lessen liver pathological damage and lower blood TG, TC, and LDL-c levels. These findings implied that DIM might lessen hepatocyte lipid buildup. AST and ALT are the most sensitive indicators of liver injury [33], and serum AST and ALT levels were increased in high-fat model mice. This study found that DIM reduced blood AST and ALT in mice fed HFD, preventing liver damage.

The ligand-activated transcription factor AhR is essential for controlling immunological responses and lipid metabolism [34]. DIM has been shown to modulate AhR signaling, thereby influencing downstream pathways involved in lipid homeostasis and inflammation [16]. MAFLD may cause liver cell fat storage and inflammation by activating p38 MAPK and increasing its phosphorylation level. Furthermore, fatty acid oxidation can be inhibited by p38 MAPK activation, which results in the buildup of liver fat [35,36]. Citrus flavonoids have therapeutic effects on lipid metabolism disorders by inhibiting the phosphorylation of MAPKs and NF-κB [37]. Our research further supported the view that DIM supplementation could restore HFD-mediated phosphorylation of p38 MAPK and elevated expression of NF-κB protein. After intervention with the AhR inhibitor CH223191 in cell experiments, the expression level of its downstream NF-κB protein was decreased. Reversal of DIM therapy boosted p38 MAPK phosphorylation and NF-κB protein expression.

Inflammation and lipid metabolism are tightly regulated by the p38 MAPK pathway. MAFLD is linked to p38 MAPK activation, which increases lipid storage and hepatic inflammation. Lipid transport proteins, including CD36, play pivotal roles in the uptake and intracellular trafficking of fatty acids [38]. Elevated expression of p38 MAPK and CD36 has been associated with increased lipid accumulation in MAFLD. Our study demonstrated that treatment with DIM effectively inhibited the phosphorylation of p38 MAPK, consequently leading to a reduction in the expression of pro-inflammatory markers such as NF-κB. This inhibition of p38 MAPK signaling is essential in reducing hepatic lipid accumulation and alleviation of liver injury, as observed in both in vivo and in vitro. Treatment of RAW 264.7 macrophages activated the p38 MAPK pathway, increased p38 MAPK phosphorylation, upregulated NF-κB expression, and boosted CD36 expression [39]. CD36, a transmembrane protein, transports extracellular long-chain fatty acids into cells for metabolism [40]. In MAFLD, p38 MAPK affects lipid uptake and metabolism by regulating the expression of CD36. CD36 is a pivotal protein in fatty acid transport, and its upregulated expression not only enhances fatty acid uptake but also exacerbates liver lipid accumulation by promoting intracellular lipid deposition [41]. In the entire animal trial, the HFD group had considerably higher p38 MAPK phosphorylation and CD36 expression. Consistent results were observed in HepG2 cells exposed to PA. DIM significantly reduced p38 MAPK phosphorylation and CD36 expression, blocking the signaling pathways. It can reduce lipid accumulation and further alleviate the progression of MAFLD, suggesting that p38 MAPK and CD36 may represent promising therapeutic targets for the management of MAFLD.

Studies have shown that the fatty acid transporter (FATP) family, particularly fatty acid transporter 4 (FATP4), is closely associated with the pathogenesis of MAFLD. Multiple metabolic organs, including the gut, liver, and adipose tissue, contain FATP4, a multifunctional protein. It predominantly transports extracellular to intracellular long-chain fatty acids. FATP4 activates fatty acids to fatty acyl-CoA, which aids intracellular fatty acid transport and metabolism and triglyceride production and storage [42]. In the MAFLD model induced by HFD, the expression level of FATP4 was significantly increased, which in turn enhanced fatty acid uptake and synthesis, ultimately leading to exacerbated fatty acid accumulation [43]. Studies have shown that CD36 and FATP4 have a synergistic effect on the function of MAFLD patients. The overexpression of CD36 results in the intracellular accumulation of fatty acids, whereas the upregulation of FATP4 influences the activation and metabolism of fatty acids. These findings have suggested that the synergistic effects of CD36 and FATP4 may lead to excessive lipid accumulation in hepatic cells [44]. Fatty acid binding protein 1 (FABP1) is extensively expressed in the kidney, small intestine, and liver, among other metabolic organs. Long-chain fatty acids are transported into cells by CD36 and FATP4, and upon binding to FABPs, they are further transported to the endoplasmic reticulum, mitochondria and nucleus for metabolism. In a rat model of fatty liver generated by HFD, FABP1 expression was dramatically increased, which impaired fatty acid metabolism and was crucial to the onset and progression of fatty liver disease [45]. A cross-sectional study found that MAFLD patients had significantly higher plasma FABP1 levels and a positive connection with body mass index and waist circumference, suggesting that FABP1 might be important in MAFLD pathogenesis [46]. Furthermore, FABP1 negatively regulated very low-density cholesterol (VLDL). Increased FABP1 expression reduced VLDL production, leading to excessive intracellular accumulation of TG and TC, which contributed to lipid metabolic disturbances [47]. Our results demonstrated that the expression level of FABP1 in HepG2 cells induced by PA was significantly increased, and DIM significantly decreased FABP1 expression. However, intervention with CH223191 could not reverse the effect of DIM, indicating that DIM reduced fatty acid transport mainly through the regulation of CD36 and FATP4.

MAFLD is most often linked to obesity and type 2 diabetes. Notably, MAFLD is prevalent in over 60% of patients with T2DM [48]. Therefore, modern clinical trials of hypoglycemic agents have increasingly focused on their effects on MAFLD. There was compelling evidence that thiazolidinediones (TZDs) and glucagon-like peptide-1 receptor agonists (GLP-1Ras) were beneficial in treating MAFLD because they improved insulin resistance, control lipid metabolism, and dramatically lower body weight [49,50,51]. The two main treatments for MAFLD are diet and exercise. Mediterranean diets have lower calories and polyunsaturated fats than Western diets. Weight and liver function improved dose–response with calorie reduction, showing that diet could improve MAFLD [52]. Furthermore, dietary artificial sweeteners have improved gut microbiota composition and diversity. Long-term use of sweeteners could diminish beneficial gut flora, causing microbial ecological imbalance and a reduction in immunity [53]. In mice, an I3C-enriched diet could normalize intestinal bacterial load, increased immunoglobulin A levels, and improve intestinal health [54]. Nutrition is vital throughout life, as it promotes linear growth and development [55] in newborns and the elderly; dietary insufficiency could cause intestinal dysfunction, inflammatory response, and immunological regulatory abnormalities [56,57]. Thus, MAFLD can be successfully prevented by maintaining a dynamic balance between growth and nutrition.

By inhibiting p38MAPK phosphorylation and suppressing fatty acid transport pathways, DIM may reduce lipid droplet accumulation and alleviate MAFLD, providing strong evidence for the use of natural phytochemicals to prevent and treat metabolic diseases. However, this work has some shortcomings that need to be investigated further. First, physiological and metabolic differences between animals and humans may limit our findings to humans. Species-specific liver metabolic pathways between mice and humans may affect the applicability of our results. Second, our data show that DIM improves MAFLD via activating AhR and inhibiting the p38 MAPK phosphorylation pathway, although the mechanisms in other tissues or cell types are unknown. The pathophysiology of MAFLD involves complex signaling pathways like oxidative stress and the gut-liver axis, making DIM’s therapeutic benefits difficult to grasp. Third, DIM has a complicated metabolic profile and poor in vivo bioavailability. We did not test DIM metabolites in mice, which may limit their therapeutic use. Research should study alternate pathways by which DIM improves MAFLD and evaluate its health effects in long-term population-based trials.

## 5. Conclusions

Taken together, the study suggests that DIM exerts significant hepatoprotective effects in MAFLD through AhR-mediated inhibition of the p38 MAPK pathway and downregulation of lipid transport proteins. These results demonstrate DIM’s potential as a therapy option for MAFLD and warrant further investigation into its clinical applications. Upcoming research initiatives should focus on elucidating the exact molecular pathways through which DIM influences AhR/p38 MAPK signaling and lipid metabolism. Additionally, long-term studies in larger cohorts of HFD-fed mice and clinical trials in human subjects are warranted further to prove DIM’s MAFLD efficacy and safety. The potential for DIM to modulate other signaling pathways and its interactions with other dietary components should also be explored to fully understand its therapeutic potential. Overall, our study underscores the importance of natural compounds in modulating key signaling pathways involved in MAFLD pathogenesis and provides a promising avenue for the development of innovative therapeutic strategies.

## Figures and Tables

**Table 1:** Differences in serum level of TC, TG, FFA, LDL-c, and HDL-c $\left(\bar{\chi }\pm s\right)$.

Item (mmol/L)	NCD	HFD	HFD + DIM
TG	0.37 ± 0.36	0.56 ± 0.62 *	0.43 ± 0.30 ^#^
TC	3.17 ± 1.48	4.58 ± 0.40 **	4.02 ± 0.33
FFA	0.58 ± 0.26	0.66 ± 0.26	0.55 ± 0.59
LDL-c	0.39 ± 0.25	0.84 ± 0.10 *	0.56 ± 0.56 ^#^
HDL-c	8.37 ± 0.23	8.16 ± 0.30	9.47 ± 0.33 *^##^

* *p* < 0.05, ** *p* < 0.01 vs. NCD group, # *p* < 0.05, ## *p* < 0.01 vs. HFD group.

**Table 2:** Differences in hepatic concentrations of TC, TG and FFA $\left(\bar{\chi }\pm s\right)$.

Item (mmol/L)	NCD	HFD	HFD + DIM
TG	17.06 ± 4.75	52.55 ± 8.78 **	24.92 ± 2.98 ^##^
TC	11.88 ± 0.62	13.09 ± 0.29	10.14 ± 0.47 *^#^
FFA	1.01 ± 1.77	1.29 ± 0.32	0.51 ± 0.77 ^#^

* *p* < 0.05, ** *p* < 0.01 vs. NCD group, # *p* < 0.05, ## *p* < 0.01 vs. HFD group.

## Data Availability

The data from this study are publicly accessible and may be requested from the corresponding author.

## Supplementary Materials

The following supporting information can be downloaded at: https://www.mdpi.com/article/10.3390/nu17101681/s1, Table S1: Antibodies.
