# Supplementary material for: 3,3′-Diindolylmethane Ameliorates Metabolism Dysfunction-Associated Fatty Liver Disease via AhR/p38 MAPK Signaling
Source: Nutrients. 2025 May 15;17(10):1681. doi: 10.3390/nu17101681 (PMC12113855; doi:10.3390/nu17101681)
Supplement: Supplementary file 1 [file nutrients-17-01681-s001.zip › nutrients-3622662-supplementary.pdf]

Table S1: Antibodies

Antibodies	Species	Dilution	Corporation
AhR	rabbit	1:1000	Abclonal, China
p-p38MAPK	mouse	1:500	Santa, USA
p38 MAPK	mouse	1:500	Santa, USA
NF-κB	rabbit	1:1000	Cell Signaling Technology, USA
CD36	rabbit	1:1000	Abclonal, China
PPARγ	mouse	1:1000	Santa, USA
FATP4	rabbit	1:1000	Abclonal, Chin
FABP1	rabbit	1:1000	Abclonal, China
β-actin	mouse	1:5000	Beijing Ray Antibody Biotech, China
